# Enhancing metabolic efficiency via novel constitutive promoters to produce protocatechuic acid in *Escherichia coli*

**DOI:** 10.1007/s00253-024-13256-6

**Published:** 2024-08-17

**Authors:** Oliver Englund Örn, Arne Hagman, Mohamed Ismail, Nélida Leiva Eriksson, Rajni Hatti-Kaul

**Affiliations:** Division of Biotechnology, Department of Chemistry, Center for Chemistry & Chemical Engineering, Lund University, Box 124, 221 00 Lund, Sweden

**Keywords:** Synthetic constitutive promoter, Inducer free expression system, Promoter library, Protocatechuic acid production, RT-qPCR

## Abstract

**Abstract:**

The antioxidant molecule protocatechuic acid (PCA) can also serve as a precursor for polymer building blocks. PCA can be produced in *Escherichia coli* overexpressing 3-dehydroshikimate dehydratase (DSD), an enzyme that catalyses the transformation of 3-dehydroshikimate to PCA. Nevertheless, optimizing the expression rate of recombinant enzymes is a key factor in metabolic engineering when producing biobased chemicals. In this study, a degenerate synthetic promoter approach was investigated to improve further the production of PCA. By limited screening of a randomized promoter library made using pSEVA221 plasmid in *E. coli*, three novel synthetic constitutive promoters were selected that increased the PCA yield from glucose by 10–21% compared to the inducible T7-promoter. RT-qPCR analysis showed that the *DSD* gene, regulated by the synthetic promoters, had high expression during the exponential phase, albeit the gene expression level dropped 250-fold during stationary phase. Besides the increased product yield, the synthetic promoters avoided the need for a costly inducer for gene expression. Screening of the entire promoter library is likely to provide more positive hits. The study also shows that *E. coli* transformed with the *DSD* gene on either pSEVA221 or pCDFDuet plasmids exhibit background PCA levels (~ 0.04 g/L) in the absence of a transcriptional regulatory element.

**Key points:**

*• Degenerate synthetic promoters are remarkable tools to produce protocatechuic acid.*

*• The constitutive synthetic promoters did not affect the growth rate of the bacterial host.*

*• The use of constitutive synthetic promoters avoids the need for the costly inducer.*

**Supplementary Information:**

The online version contains supplementary material available at 10.1007/s00253-024-13256-6.

## Introduction

Metabolic engineering requires several parameters working in unison to maximize the production of a desired metabolite (Andreozzi et al. 2016; Averesch et al. 2018; Skoog et al. 2018; Yim et al. 2011). Flux control analysis of metabolic pathways shows that the control is distributed along all parts of the pathway and no single factor dominates alone. With that in mind, a factor that has been shown to be an effective target is the level of critical enzyme(s) in the cell. Tuning the enzyme concentration is possible in many ways, one of them being through optimizing the transcription efficiency of the gene encoding the enzyme (Engstrom & Pfleger 2017). Not having enough active enzyme can create a bottleneck, while too high expression can put an unnecessary metabolic burden on the cell (Zhou et al. 2011). An approach used often to optimize the enzyme levels is to make use of inducible promoters, which allows fine tuning of gene expression levels, but these systems have limitations when scaling up in terms of inducer costs, hypersensitivity to inducer concentration, and transcriptional heterogeneity between cells (Mnaimneh et al. 2004; Siegele & Hu 1997). Moreover, the individual expression of several genes cannot be fine-tuned with inducible promoters unless different inducers are used for each gene.

In *Escherichia coli*, transcription requires recognition of the promoter by a σ-factor of the RNA polymerase (RNAP). σ^70^ is the factor most used in *E. coli* for initiation of most genes (Lonetto et al. 1992); it recognizes a consensus region about 35 bp in length that consists of –10 and –35 elements (TATAAT and TTGACA, respectively), the numbering corresponding to their spacing relative to the first transcribed base (+ 1) (Engstrom and Pfleger 2017). The sequence and hence structure of the promoter control how strong and how often the RNAP binds to the DNA and can be used to control transcription rates (Engstrom & Pfleger 2017). Using a defined promoter library, one can set the transcription level for each gene, but this approach is work intensive, as a new construct needs to be built for each tested promoter. However, employing a modular cloning strategy can help speed up the process (Iverson et al. 2016). Another approach is the one reported earlier (De Mey et al. 2007), in which a 57-bp-long degenerate synthetic promoter, consisting of a sequence of 24 conserved, 13 semi-conserved (W = A or T, R = A or G, D = A, C or G) and 20 random (N) nucleotides was arranged to fit to a consensus promoter in *E. coli* (Table 1). With the degenerate synthetic promoter, a nearly linear increase in expression rate was observed among the resulting new promoters. Therefore, if the optimal expression rate to observe the phenotype caused by the gene of interest is unknown, this degenerate synthetic promoter can be used to control the gene of interest and the optimal expression rate can be selected.

**Table 1:**
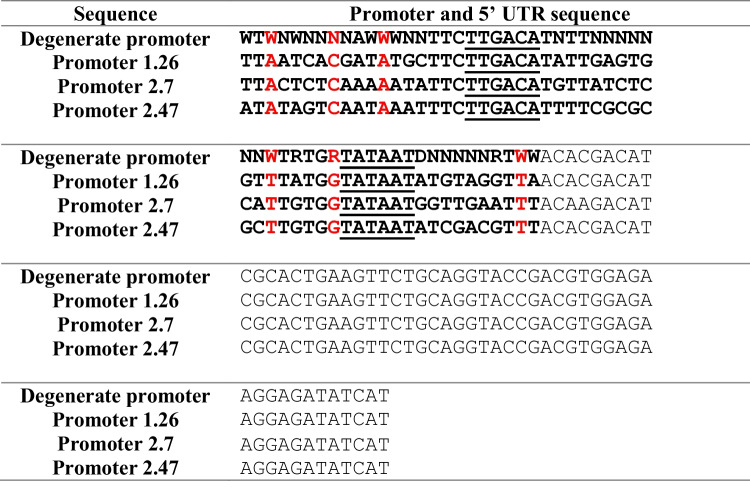
Sequence alignment of the promoter with degenerate bases and the three characterized promoters. The promoter sequence is in bold, the 5’UTR is in normal font, the − 35 and − 10 sequences are underlined, and the conserved degenerate bases are highlighted in red. Degenerate base description: W = A or T, R = A or G, D = A, C or G and N = A, T, C or G

This study is a continuation of our earlier work in which the production of protocatechuic acid (3,4-dihydroxybenzoic acid) (PCA) from glucose was investigated using an *E. coli* strain ATCC 31882 (a K12-derived strain that overproduces phenylalanine) (US Patent 4,681,852) engineered with the gene 3-dehydroshikimate dehydratase (*DSD*) on a pCDFDuet plasmid and controlled by T7-promoter (Örn et al. 2021). PCA is a chemical with antioxidant properties and is used as an anti-inflammatory agent (Kakkar & Bais 2014). It is also an important intermediate to produce several aromatic molecules and C6 aliphatic molecules (Pugh et al. 2014; Weber et al. 2012). In the present work, degenerate synthetic promoters from a randomized library were introduced in a single cloning step upstream of the *DSD* gene to find an optimal transcription rate and thereby bypassing the use of an inducer like isopropyl β-D-1-thiogalactopyranoside (IPTG) for gene expression. The study further investigates the unexpected functionality of the T7-promoter in the pCDFDuet plasmid when transformed into the ATCC 31882 strain lacking T7-RNAP (Örn et al. 2021).

## Methods

### Plasmid construction

The *DSD* gene (UniProt ID: Q88JU3) (abbreviated in other organisms as *3dhsd*, *AroZ*, *AsbF*, and *QuiC1*) was amplified from genomic DNA of *Pseudomonas putida* using the primers listed in Table S1. The genomic DNA was purified with E.Z.N.A bacterial DNA kit (OMEGA Bio-Tek, Norcross, GA, USA). The pCDFDuet-1 plasmid was purchased from Novagen (EMD Chemicals, CA, USA). The low copy number plasmid pSEVA221, transformed into *E. coli*, was a gift from Prof. Victor de Lorenzo’s Lab (Centro Nacional de Biotecnología, Spain) (Martínez-García et al. 2015). *E. coli* containing either pCDFDuet or pSEVA221 was grown in LB medium with 50 mg/L streptomycin or kanamycin. The *E. coli* strain containing the relevant plasmid was grown overnight in at least 10 mL culture volume and the plasmid extracted using GeneJet plasmid purification kit (Thermo Scientific, Waltham, MA, USA).

To construct the promoter library, the pSEVA221 plasmid was linearized using FastDigest restriction enzymes EcoRI and KfII (Thermo Scientific) and then purified by gel extraction using QIAquick Gel Extraction Kit (QIAGEN, Hilden, Germany). The synthetic terminator B0015 was purchased in two parts, each 138 bp long, as single-stranded (ss) DNA (Eurofins Genomics, Ebersberg, Germany). The degenerate promoter sequence based on *E. coli* consensus promoters (De Mey et al. 2007), including the 5′UTR and overhangs complementary to the plasmid for the assembly was also purchased as a synthetic ssDNA. All other synthetic DNA fragments were purchased as dsDNA from IDT (Coralville, Iowa, USA). The ssDNA fragments were made double-stranded (ds) in a two-cycle PCR reaction with the primers described in Table S1. All PCR reactions were performed with Q5 High-Fidelity DNA Polymerase (New England Biolabs, Ipswich, MA, USA) according to the manufacturer’s specification. The four fragments used for the construction of the plasmid library pSEVA221-*DSD*, i.e., linearized pSEVA221 plasmid, *ds* terminator, *ds* degenerate synthetic promoter, and *DSD* gene, were assembled using NEBuilder (New England Biolabs) according to the manufacturer’s specifications. It should be mentioned that the ssDNA fragments were also tested for a one-step assembly, but with negative results. Instead, the ssDNA fragments had to be converted to dsDNA, prior to the assembly for higher efficiency.

**Fig. 1:**
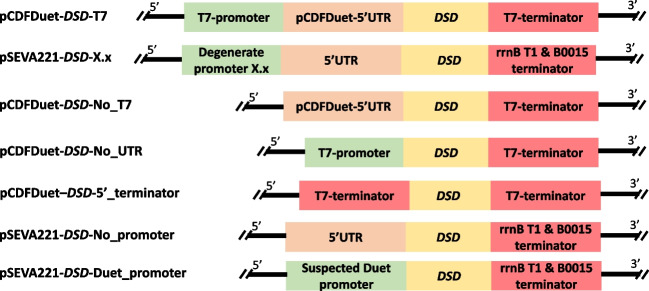
Schematic representation of all plasmids used in the study and the respective regulatory elements for the gene of interest *DSD*. Each plasmid name is defined by the convention: vector-*gene of interest*-regulatory element at the 5 ′ end of the gene of interest. In pSEVA221-*DSD*-X.x, the X.x is replaced by a number for each unique degenerate promoter sequence. In pCDFDuet-*DSD*-No_T7, the T7 promoter upstream of DSD gen (19 bp) was deleted. In pCDFDuet-*DSD*-No_UTR, the UTR upstream of DSD gene (110 bp) was removed. In pCDFDuet-*DSD*-5′_terminator, the double terminators rrnB T1 and T7Te replaced the T7-promoter and 5′UTR upstream of *DSD* gene. In pSEVA221-*DSD*-No_promoter, the degenerate synthetic promoter sequence upstream of *DSD* gene was removed. In pSEVA221-*DSD*-Duet_promoter, the degenerate promoter and 5′UTR were replaced by the suspected Duet promoter region found by iPromoter-2L (Liu et al. 2018)

All other plasmids that did not contain the degenerate promoter (Fig. 1) were constructed by replacing fragments of the pSEVA221-*DSD-1.26* or pCDFDuet-*DSD-*T7 plasmids with synthetic dsDNA fragments, purchased as dsDNA from IDT, using NEBuilder. pCDFDuet-*DSD-*T7 and pSEVA221-*DSD*-1.26 were linearized with the restriction enzyme MluI/BamHI or NotI/PflFI. The linearized plasmid backbone was gel purified with QIAquick Gel Extraction Kit and assembled by NEBuilder with the synthetic dsDNA according to manufacturer’s specification before transformation into *E. coli* DH5α.

### Construction of recombinant *E. coli* strains

The constructed promoter library was transformed into competent *E. coli* DH5α cells. From these, 180 unique DH5α colonies were picked and stored as glycerol stocks at − 80°C and the plasmids were purified and pooled together. Then, the plasmids purified from the DH5α colonies were transformed into competent *E. coli* ATCC 31182 (US Patent 4,681,852). One hundred and eighty new colonies were picked and screened for PCA production (as described below). All other plasmids (Fig. 1) were transformed into either of the three following competent *E. coli* strains*:* ATCC 31182, BL21(DE3), or BW25113. *E. coli* ATCC 31182-pCDFDuet-*DSD-T7* and *E. coli* BL21(DE3)-pCDFDuet-*DSD-T7* were used in our earlier study for PCA production (Örn et al. 2021).

### Growth conditions of *E. coli* strains and PCA production

*E. coli* ATCC 31882 was used to screen PCA production with the constructed degenerate synthetic promoter library in the plasmid pSEVA221-*DSD*. The transformed colonies were grown on selective medium containing 50 mg/L kanamycin or streptomycin and inoculated into 100 µL LB medium (Invitrogen, Waltham, MA, USA). The cells were allowed to grow for 4 h at 37 °C in a 96-well microtiter plate (Sarstedt, Nümbrecht, Germany). Fifty microliters of the LB cultures was then used to inoculate 1 mL modified M9 medium in triplicate in 96-deep well plates (Sarstedt). The modified M9 medium contained per liter: 8.5 g Na_2_HPO_4_·2H_2_O, 3 g KH_2_PO_4_, 0.5 g NaCl, 2 g NH_4_Cl, 1 mg thiamine, 2 mM MgSO_4_, 0.1 mM CaCl_2_, 28 µg FeSO_4_, 36 µg (NH_4_)_6_Mo_7_O_24_·4H_2_O, 248 µg H_3_BO_3_, 72 µg CoCl_2_, 24 µg CuSO_4_, 160 µg MnCl_2_, 28 µg ZnSO_4_, 40 mg tryptophan, 40 mg tyrosine, and 35 g glucose, unless otherwise specified. The plates were sealed with an air permeable 114-µm Rayon film (VWR, Radnor, PA, USA), and grown at 37 °C, 800 rpm for 48 h. To compare PCA production levels, the following controls were included in the screening experiments: *E. coli* BL21(DE3), *E. coli* ATCC 31182, and *E. coli* BW25113 transformed with the plasmids pCDFDuet-*DSD-*T7, pCDFDuet*-DSD-*No_T7, pCDFDuet*-DSD-*No_UTR, pCDFDuet*-DSD-*5'_terminator, pSEVA221-*DSD*-No_promoter, and pSEVA221-*DSD*-Duet_promoter induced (with 1 mM IPTG) and non-induced. The empty strains of *E. coli* BL21(DE3), *E. coli* ATCC 31182, and *E. coli* BW25113 were also included as controls.

The positive hits from the library screening as well as *E. coli* BL21(DE3)-pCDFDuet-*DSD-T7*, *E. coli* ATCC 31182-pCDFDuet-*DSD-T7*, ATCC 31882-pSEVA221-*DSD*-1.77, ATCC 31882-pCDFDuet-*DSD*-No_T7, ATCC 31882-pSEVA221-*DSD*-No_promoter, and *E. coli* ATCC 31182 were grown in duplicates in baffled 500-mL shake flasks containing 150 mL modified M9 medium at 37 °C, 200 rpm for 48 h. The selective medium was supplemented with 50 mg/L of either kanamycin or streptomycin for the pSEVA221 and the pCDFDuet plasmid, respectively.

Growth of *E. coli* ATCC 31882-pSEVA-*DSD*-1.26 was also performed in a 3-L bioreactor (Applikon Biotechnology, Delft, The Netherlands) containing 1 L modified M9 medium at 37 °C and the DOT controlled above 40% by the stirring rate and an air flow of 1 vvm. The pH was controlled at pH 7 by addition of 1 M NaOH.

### Analytical methods

Cell growth was monitored by measuring OD at 600 nm of 1-mL samples using a 1-cm wide cuvette in a Lambda Bio + spectrophotometer (PerkinElmer, Waltham, MA, USA). For the cultivations done in a 96 well-plate format, the OD_600_ was determined in 100-µL samples using a Multiskan GO plate-reader (ThermoFischer). A standard curve was made to correlate the measurements from the plate reader to the cuvette due to the difference in path lengths between the two machines. The conversion of measured OD_600_ values to cell dry weight (CDW) and conversion into cmol were performed according to the method described earlier (Örn et al. 2021). Similarly, the concentrations of PCA, glucose, and organic acids, in grams per liter, were measured using HPLC according to the published protocols (Örn et al. 2021). The PCA yield with respect to glucose consumed (*Y*_p/s_), biomass (*Y*_p/b_), and acetate yield from glucose (*Y*_a/s_) were calculated in mol/mol or cmol/cmol based on the final concentration of each metabolite. The PCA productivity was calculated based on the total PCA concentration at 36 h. For all samples where replicates were measured, the standard deviation between replicates was calculated.

Soluble and insoluble protein fractions from *E. coli* ATCC 31882 were obtained by sonicating cell samples from the shake flask cultivation for 2 min at 24 kHz in a sonicator (Hielscher GmbH, UP 400) and visualized on SDS-PAGE with a 4–20% Mini-PROTEAN® TGX Stain-Free™ Protein Gel (Bio-Rad, Hercules, CA, USA). To determine the molecular weight, Precision Plus Protein Unstained Protein Standards (Bio-Rad) was used as reference.

### RT-qPCR analysis

Total RNA was extracted from *E. coli* ATCC 31882 strains using *TRIzol*™ Reagent (Invitrogen) according to manufacturer’s instructions. Any residual DNA in the RNA sample was degraded by adding 1 µL ezDNAse (Invitrogen) per 10 µg total RNA and incubated for 2 min at 37 °C. cDNA was amplified from the ezDNAse treated total RNA using 100 U Maxima H Minus Reverse Transcriptase (Thermo Scientific), and 100 pmol ReadyMade Randomers (IDT), according to the protocol of the transcriptase for random hexamer primers. The quality of cDNA was verified by measuring the 260/280 nm 260/230 nm ratios in a BioSpec-nano spectrophotometer (Shimadzu, Kyoto, Japan) and values above 1.8 were accepted. qPCR was performed with 100 ng cDNA as template using primers listed in Table S1 and the PowerTrack™ SYBR Green Master Mix (Applied Biosystems, Waltham, MA, USA) in a total volume of 10 µL. The RT-qPCR protocol started with initial denaturation at 95 °C for 2 min, followed by 40 cycles of 15 s at 95 °C and 1 min at 60 °C. At the end of each cycle, the fluorescent signal from SYBER Green was measured. This was followed by a final extension of 5 min at 72°C and melt curve determination. The RT-qPCR was performed using a CFX96 Touch Real-Time PCR detection system (Bio-Rad), and the data was analyzed with CFX Maestro (Bio-Rad), which determined the quantification cycle values (*C*_q_). For each measurement, two biological replicates and four technical replicates were made. For each biological replicate, a negative RT sample was run to determine potential gDNA contamination. To determine the relative quantification cycle, (Δ*C*_q_) of *DSD* for each promoter and time point the housekeeping genes *cysG*, *hcaT*, and *idnT* were used as reference. These genes have been shown to be stably expressed when overexpressing proteins in *E. coli* (Zhou et al. 2011), and the stability was verified in the generated dataset in CFX Maestro. To determine the fold change in expression of *DSD* between the constitutive and inducible promoters, the 2^−ΔΔCq^ method was used (Livak and Schmittgen 2001).

## Results

### Screening of PCA production in promoter library

**Fig. 2:**
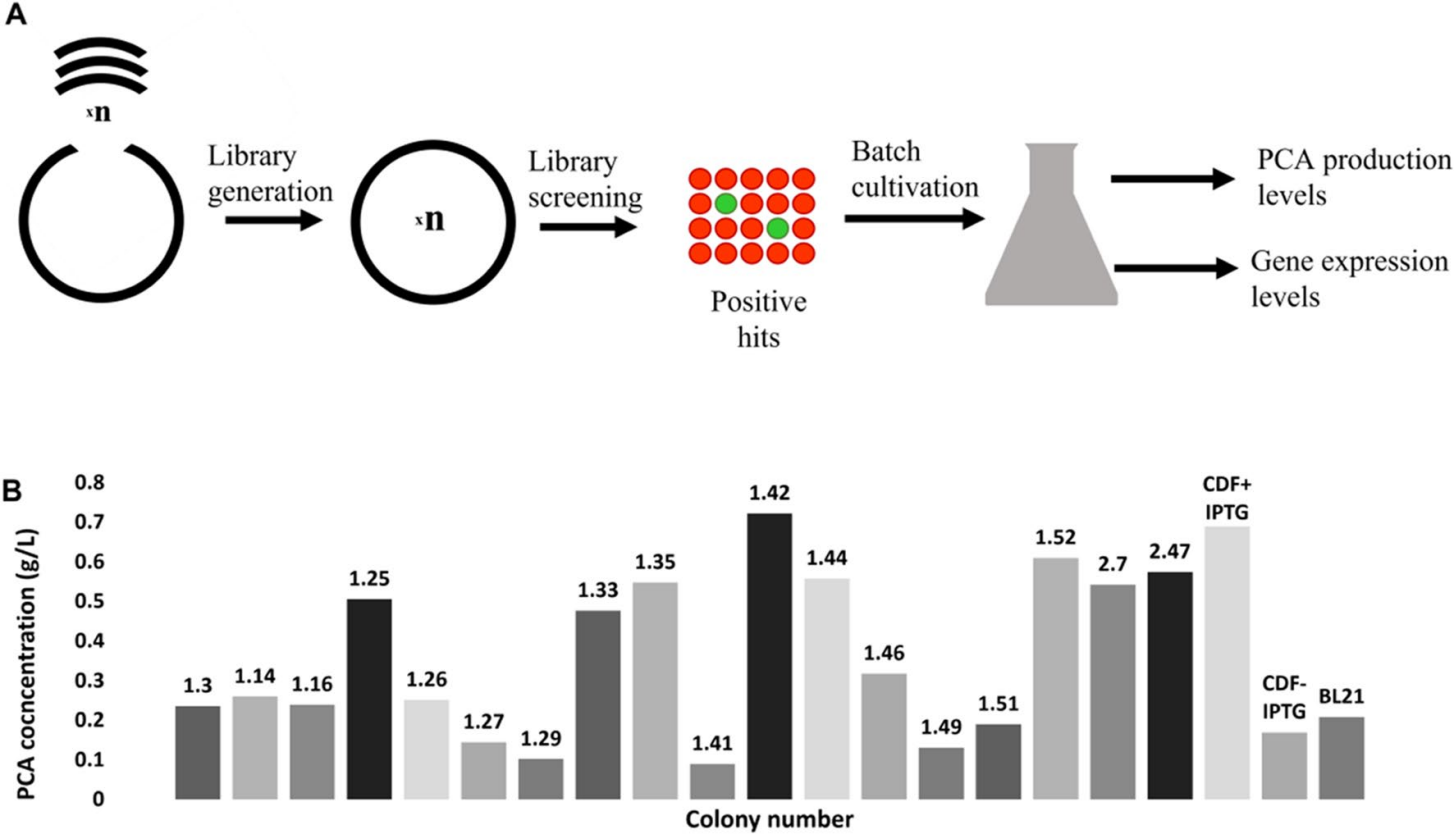
**A** Overview of degenerate promoter library workflow for PCA production. A promoter library was generated and screened for PCA production in the *E. coli* strain ATCC 31882. The positive hits were cultivated in a batch reaction and the PCA production levels and gene expression levels of *DSD* were determined in each strain with a unique constitutive promoter. **B** PCA titers of the positive hits from the ATCC 31882 pSEVA221-*DSD* library screening, as well as the controls of IPTG induced (CDF + IPTG) and non-induced (CDF-IPTG) ATCC 31882-pCDFDuet-*DSD-*T7, and BL21(DE3) -pCDFDuet-*DSD-*T7 (BL21). The controls and colonies 1.26, 2.7, and 2.47 produced PCA in all three replicates, the remaining produced PCA in only one replicate

We constructed a promoter library in *E. coli* DH5α using one plasmid assembly step. The degenerate synthetic promoter sequence was designed as previously described (De Mey et al. 2007) (Fig. 2A). The library was constructed in the low copy plasmid pSEVA221 which has a copy number of 1–2 per cell to mimic the conditions after genomic integration (Martínez-García et al. 2015). One hundred and eighty randomly selected plasmids from the DH5α library were transformed into the production strain *E. coli* ATCC 31182. From an initial screening of the 180 colonies, only three were found to consistently produce PCA in all three biological replicates; they were named 1.26, 2.7, and 2.47. The numbering is based on the plate and colony number, and the number is added to the end of the plasmid name with a dash to signify which promoter is used. Fifteen other colonies produced PCA in only one replicate (Fig. 2B), while the other 162 colonies did not reveal any detectable production of PCA. The average PCA production after 48 h in colonies 1.26, 2.7, and 2.47 were 0.25 ± 0.13 g/L, 0.54 ± 0.32 g/L, and 0.57 ± 0.30 g/L, respectively. Even though promoters like 1.42, 1.52, and 1.35 exhibited initial high PCA production, the result was not consistent in all three replicates, indicating that they are less stable in their behavior.

On the other hand, *E. coli* ATCC 31182 transformed with pCDFDuet-*DSD-T7* construct, and either induced with 1 mM IPTG or un-induced resulted in the production of 0.689 ± 0.028 g/L and 0.169 ± 0.025g/L PCA, respectively. However, *E. coli* BL21(DE3) transformed with pCDFDuet-*DSD*-T7 induced with 1 mM IPTG produced 0.208 ± 0.058 g/L PCA but did not produce any PCA without induction.

Table 1 lists the sequences of the degenerate synthetic promoter and the three characterized promoters 1.26, 2.7, and 2.47 obtained by Sanger sequencing. The sequences of promoters 1.26, 2.7, and 2.47 were blast searched and no significantly similar sequences were found in the NCBI Nucleotide-BLAST online database. Of the 33 degenerate bases, six were conserved in all three promoters, i.e., 18% sequence identity.

### PCA production with plasmids having altered transcription regulatory elements

To elucidate PCA production by the pCDFDuet plasmid in *E. coli* strains, we tested the expression of *DSD* in plasmids with added or removed regulatory elements (promoter, 5′UTR, or terminator) (Fig. 1). We also wanted to elucidate if the part of the pCDFDuet’s 5′UTR sequence could by itself promote transcription of the *DSD* gene. Using the software iPromoter-2L (Liu et al. 2018), a region in the pCDFDuet plasmid downstream of the T7-promoter up to the start of *DSD* was identified as a potential promoter sequence that could be recognized by σ^70^ RNAP (named, Duet_promoter). This sequence (Table S1) was cloned into the pSEVA221-*DSD* plasmid (pSEVA221-*DSD*-Duet_promoter) by replacing the synthetic promoter sequence 1.26. A complete description of the plasmids including the changed regulatory element can be seen in Fig. 1. The production of PCA using the synthetic promoters was also added to these experiments for comparison.

**Fig. 3:**
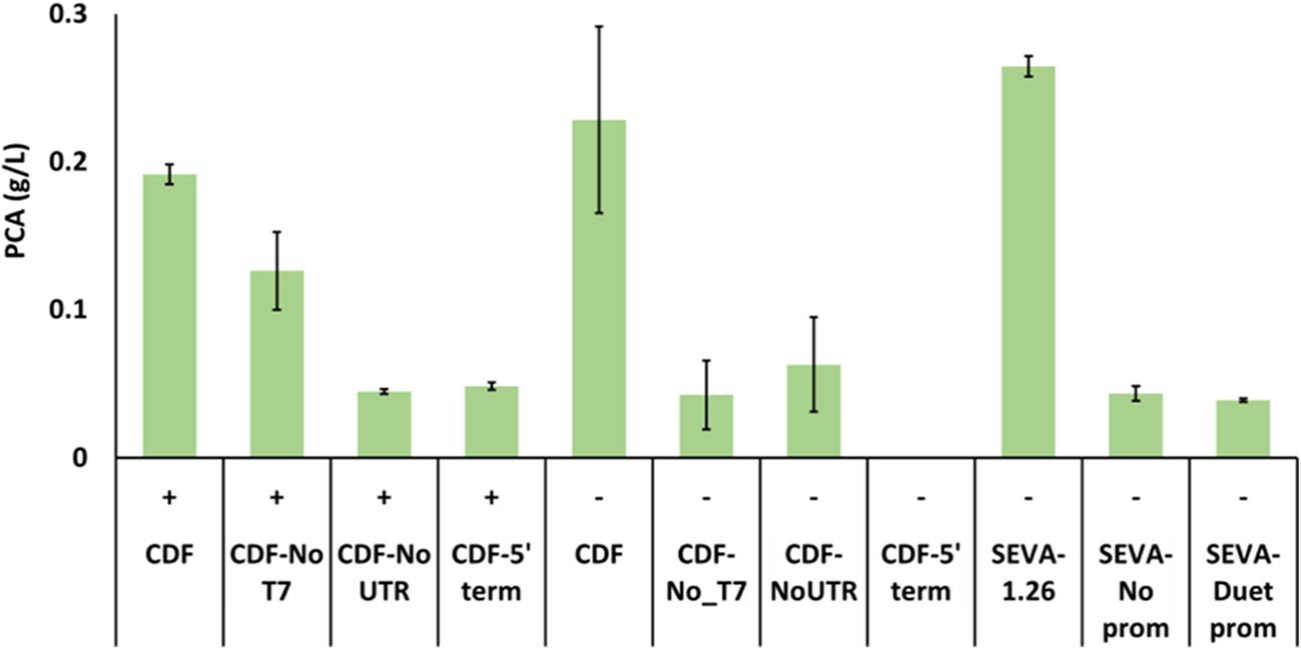
PCA titers in the 48-h culture of *E. coli* ATCC 31882. The cells were transformed with the plasmids, pCDFDuet-*DSD-*T7 (CDF); pCDFDuet-*DSD*-No_T7 (CDF-No T7); pCDFDuet-*DSD*-No_UTR (CDF-No_UTR); pCDFDuet-*DSD*-5′_terminator (CDF-5′_term); pSEVA221-*DSD*-1.26 (SEVA-1.26); pSEVA221-*DSD*-No_promoter (SEVA-No prom); and pSEVA221-*DSD*-Duet_promoter (SEVA-Duet prom). The cells were grown in 1 mL modified M9 medium in a 96-deepwell plate in the presence ( +) or absence (–) of 1 mM IPTG in the cultivation medium. All strains were cultivated in triplicate and error bars represent the standard deviation

Plasmids pCDFDuet-*DSD*-T7, pCDFDuet-*DSD*-No_T7, pCDFDuet-*DSD*-No_UTR, pCDFDuet -*DSD*-5'_terminator, pSEVA221-*DSD*-No_promoter, and pSEVA221-*DSD*-Duet_promoter were each cloned into *E. coli* ATCC 31182, BL21(DE3), and BW25113. The transformed strains were grown in 1 mL modified M9 medium in 96-deepwell plates for 48 h with and without addition of 1 mM IPTG after which PCA concentrations were measured. The constructs in both *E. coli* BW25113 (Fig. S2A) and BL21(DE3) (Fig. S2B) exhibited very low to no production levels of PCA compared to the ATCC 31182 strain (Fig. 3).

In *E. coli* ATCC 31182, comparable levels of PCA production were observed in the presence and absence of IPTG when the regulatory elements in the pCDFDuet vector were intact, i.e., pCDFDuet-*DSD*-T7 (CDF in Fig. 3); similar results were observed when 19 bp of the T7-promoter was removed and IPTG was added, i.e., cells transformed with pCDFDuet-*DSD*-No_T7 (CDF-No T7 in Fig. 3). These results indicate that the 19-bp T7-promoter by itself is not necessary for the expression of *DSD* and hence the production of PCA. But keeping the entire regulatory element intact is beneficial for PCA production, as observed by the low PCA production in the cells that had modified regulatory elements, i.e., pCDFDuet-*DSD*-No_UTR (CDF-NoUTR in Fig. 3) and pCDFDuet -*DSD*-5′_terminator (CDF-5′term in Fig. 3).

When the promoter region of the pCDFDuet (Table S1) predicted by the online promoter prediction tool iPromoter-2L (Liu et al. 2018) was used to promote transcription of *DSD* in pSEVA221, it resulted in the production of PCA at concentrations similar to those obtained in cells containing the *DSD* gene but without a promoter, i.e., pSEVA221-*DSD*-No_promoter (SEVA-No prom in Fig. 3). This result led us to conclude that the predicted promoter region was not relevant for *DSD* expression.

The most significant result was the discovery that the synthetic promoter 1.26 not only produced the highest concentration of PCA but also did it in a more consistent manner as indicated by the narrow standard deviation (Fig. 3). On the other hand, when *DSD* is under the control of the T7-promoter, no significant differences in PCA production were observed in the presence or absence of IPTG although greater variability was observed when IPTG was absent.

**Fig. 4:**
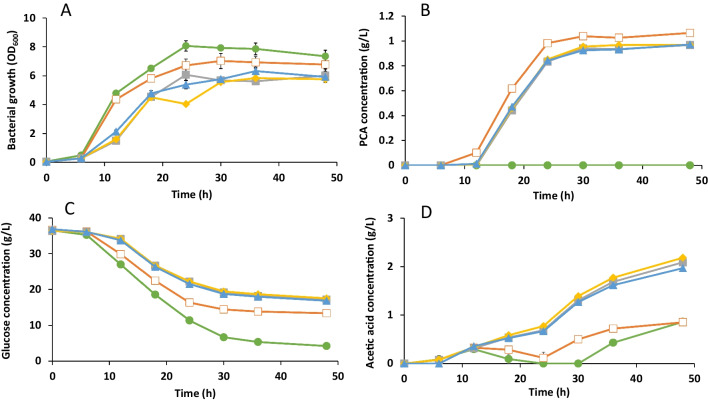
Profiles of **A** bacterial growth, **B** PCA concentration, **C** glucose concentration, and **D** acetic acid concentration during cultivations of ATCC 31882 (circle); ATCC 31882-pCDFDuet-*DSD*-T7 (empty square); ATCC 31882 pSEVA221-*DSD*-2.7 (filled square); ATCC 31882 pSEVA221-*DSD*-1.26 (diamond), and ATCC 31882 pSEVA221-*DSD*-2.47 (triangle) in 150 mL modified M9 medium in shake flasks. All strains were cultivated in duplicates and error bars represent the standard deviation

A generally accepted concept is that inducible promoters are more suitable for addressing metabolic burdens. However, the results observed in Figs. 3 and 4 show that the constitutive synthetic promoters, created in this study, do not represent a greater load (as seen by the rate of bacterial growth) compared to wild-type cells or those carrying the inducible promoter T7. Instead, comparable production of PCA is observed without the use of an expensive inducer which would reduce production costs in a large-scale process.

### Shake flask cultivations

To validate the results from 1-mL cultivations (Figs. 2 and 3), the three constructs with synthetic promoters that showed consistent PCA production during screening were scaled up to 150 mL together with two controls, wild-type *E. coli* ATCC 31182 and *E. coli* ATCC 31182-pCDFDuet-*DSD*-T7 (with inducible T7-promoter). The PCA production parameters for all the strains are listed in Table 2. The three cultures with unique synthetic promoters (plasmids pSEVA221-*DSD-*1.26, 2.7, and 2.47) exhibited similar PCA titer and PCA yield with respect to glucose consumed (*Y*_p/s_) and biomass formed (*Y*_p/b_). Cell growth, glucose consumption, and acetate formation profiles were also similar in the three cultures (Fig. 4 and Table 2). In comparison to *E. coli* ATCC 31182-pCDFDuet-*DSD*-T7 with the inducible T7 promoter, the cell growth (*p*-value 0.17 compared to pSEVA221-*DSD-*1.26), glucose consumption (*p*-value 0.001), and PCA titer (*p*-value 0.04) were higher, and *Y*_p/s_ was between 10 and 21% higher with 1.26 having the highest yield of 0.064 at 36 h.

**Table 2:** Cell dry weight (*CDW*), PCA titer, and maximal PCA yield with respect to glucose (*Y*_p/s_) and biomass (*Y*_p/b_), and acetate yield with respect to glucose (*Y*_a/s_) at 36 h of cultivation in shake flasks with wild-type *E. coli* ATCC 31882 cells (ATCC 31882), cells with inducible DSD expression (ATCC 31882 pCDFDuet-*DSD-*T7), cells with constitutive DSD expression (ATCC 31882 pSEVA221-*DSD*-2.7, 1.26, 2.47, and 1.77), and cells without promoter (pCDFDuet-*DSD*-No-T7 and pSEVA221-*DSD*-No_promoter). Cultivation of ATCC 31882 pSEVA221-*DSD*-1.26 in a 3-L bioreactor is also described. For all strains with a pCDFDuet plasmid, 1 µM IPTG was added. All strains were cultivated in duplicates and the standard deviation was calculated

Strain	CDW (g/L)	PCA titer (g/L)	*Y*_p/s_ (mol/mol)	*Y*_p/b_ (cmol/cmol)	*Y*_a/s_ (mol/mol)	Productivity (g/L/h)
ATCC 31882	4.27 ± 0.22	NA	NA	NA	0.095 ± 0.0007	NA
ATCC 31882 pCDFDuet-*DSD-T7*	3.76 ± 0.22	1.06 ± 0.012	0.053 ± 0.0008	0.28 ± 0.017	0.28 ± 0.0056	0.028 ± 0.00024
ATCC 31882 pSEVA221-*DSD*-2.7	3.05 ± 0.04	0.97 ± 0.0044	0.060 ± 0.0002	0.32 ± 0.023	0.30 ± 0.0054	0.026 ± 9.15E − 05
ATCC 31882 pSEVA221-*DSD*-1.26	3.17 ± 0.13	0.97 ± 0.015	0.064 ± 0.0009	0.32 ± 0.008	0.25 ± 0.0023	0.027 ± 0.00031
ATCC 31882 pSEVA221-*DSD*-2.47	3.44 ± 0.14	0.97 ± 0.0017	0.058 ± 0.0001	0.28 ± 0.016	0.26 ± 0.0016	0.026 ± 3.58E − 05
BL21(DE3) pCDFDuet-*DSD-T7*	6.79 ± 0.16	0.56 ± 0.042	0.027 ± 0.0034	0.084 ± 0.0039	0.26 ± 0.0071	0.014 ± 0.00087
ATCC 31882 pSEVA221-*DSD*-1.77	1.90 ± 0	0.048 ± 0.022	0.0027 ± 0.00013	0.021 ± 0.013	0.19 ± 0.031	0.0010 ± 0.00046
ATCC 31882 pCDFDuet-*DSD-*No_T7	4.18 ± 0.25	0.90 ± 0.051	0.035 ± 0.023	0.23 ± 0.0029	0.07 ± 0.016	0.023 ± 0.0011
ATCC 31882 pSEVA221-*DSD* No_promoter	4.68 ± 0	0.072 ± 0.008	0.0026 ± 0.00025	0.018 ± 0.0019	0.046 ± 0.0046	0.0015 ± 0.00016
ATCC 31882 pSEVA-*DSD*-1.26 in a bioreactor	3.54 ± 0.081	2.47 ± 0.62	0.081 ± 0.022	0.72 ± 0.15	0.29 ± 0.046	0.069 ± 0.017

The control strain BL21(DE3) pCDFDuet*-DSD* induced with IPTG produced 47% less PCA (0.560 g/L) compared to the same plasmid in ATCC 31882 (*p*-value 0.042), while one of the negative hits from the screening, strain ATCC 31882 pSEVA221-*DSD*-1.77, revealed trace amounts of PCA (0.048 g/L) in the medium at a 150-mL scale. In general, the PCA production increased in all strains in the 150-mL scale compared to the 1-mL cultivations.

Two plasmids without a promoter at the 5′ end of *DSD* (i.e., pCDFDuet-*DSD*-No_T7 and pSEVA221-*DSD*-No_promoter) were created as controls for the novel synthetic promoters to check if another promoter sequence on the plasmid could lead to the expression of the *DSD* gene. This was especially interesting in the case of pCDFDuet plasmid expressed in the *E. coli* ATCC 31882 strain which lacks the T7-RNAP. When the 19-bp T7-promoter sequence was removed from the pCDFDuet-*DSD* plasmid (i.e., in ATCC 31882 pCDFDuet-*DSD*-No_T7), the production of PCA was not eliminated, but only slightly lowered by 15% to 0.90 g/L compared to the 1.06 g/L in ATCC 31882 pCDFDuet-*DSD*-T7 (*p*-value 0.008). However, removing the 57-bp-long constitutive promoter sequence, i.e., ATCC 31882 pSEVA221-*DSD-*No_promoter, reduced the PCA concentration by 93% to 0.072 g/L compared to the novel synthetic promoter pSEVA221-*DSD*-1.26 (*p*-value 0.002) (Table 2).

### Bioreactor cultivation

**Fig. 5:**
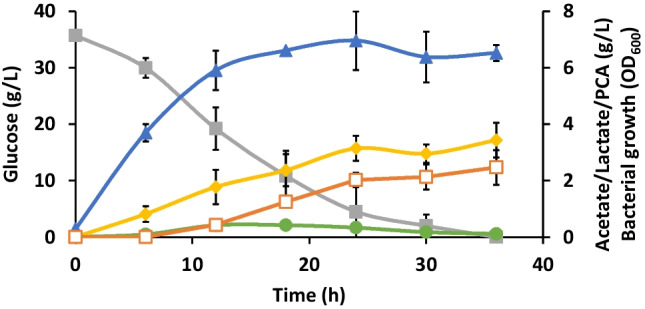
Profiles of bacterial growth (OD_600_) (triangle), glucose (filled square), acetate (diamond), lactate (circle), and PCA (empty square) concentrations during a 1-L batch cultivation of *E. coli* strain ATCC 31882 pSEVA221-*DSD*-1.26 in a 3-L bioreactor with a modified M9 medium. The experiment was performed in duplicates and error bars represent standard deviation

The strain with the highest PCA yield among the novel synthetic promoters, ATCC 31882 pSEVA221-*DSD*-1.26, was cultivated in a 3-L bioreactor, as changes in the cultivation conditions were shown to impact PCA titers in the previous study (Örn et al. 2021). Switching to a bioreactor increased the PCA titer to 2.47 g/L at 36 h with a yield on glucose of 0.081 and productivity of 0.069 g/L^.^h (Fig. 5). This is among the highest reported concentration of PCA achieved in a batch cultivation (Örn et al. 2021). However, the acetate level was increased with a yield of 0.29 mol/mol with respect to the substrate (Table 2, Fig. 5), which is most likely the cause for lower growth and glucose consumption in strains with the synthetic promoters. However, having lower glucose consumption and growth rate did not show any effect on PCA production compared to the T7 induced system.

### Expression levels of DSD in *E. coli* strains

**Fig. 6:**
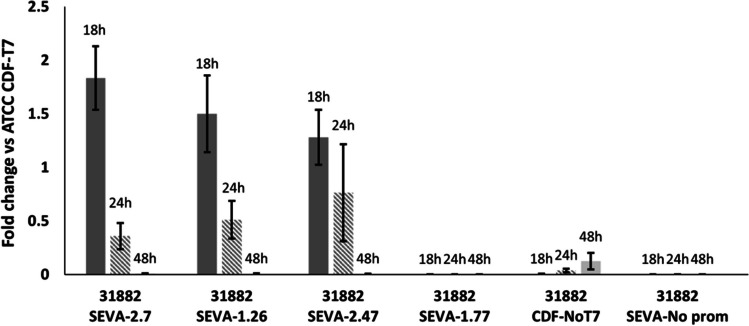
Fold change in expression of *DSD* in *E. coli* ATCC 31882 determined by RT-qPCR. The *DSD* gene was under the control of the synthetic constitutive promoters -in pSEVA221-*DSD-*2.7, 1.26, 2.47, or 1.77 (SEVA-2.7, SEVA-1.26, SEVA-2.47, and SEVA-1.77, respectively). The “promoterless” plasmids, pCDFDuet-No_T7 (CDF-NoT7) and pSEVA221-*DSD*-No_promoter (pSEVA-No_prom), are also shown. The expression is normalized against the expression of *DSD* in *E. coli* ATCC 31882 pCDFDuet-*DSD-*T7 induced by 1 mM IPTG, at mid-exponential phase (18 h) (dark gray), late exponential phase (24 h) (checkered pattern), and late stationary phase (48 h) (light gray)

Expression of the *DSD* gene in *E. coli* strains with the synthetic promoters was studied at different phases of growth, normalized by expression in ATCC 31182 pCDFDuet-*DSD*-T7 induced by 1 mM IPTG. The highest level of expression was observed at 18 h of growth for pSEVA221-*DSD*-2.7 followed by 1.26 and 2.47. Interestingly, the transcription level of *DSD* under the control of synthetic promoters was not constant throughout the cultivation period (Fig. 6). A decrease in the expression of *DSD* by the synthetic promoters was seen between the mid-exponential phase (18 h) and the beginning of the stationary phase (24 h) compared to the control pCDFDuet-*DSD* (Fig. 6). This is partly attributed to the increase in mRNA amounts in the control pCDFDuet*-DSD*-T7 during that period (Table S3). However, at the end of the stationary phase (48 h), the gene expression in pSEVA221-*DSD*-1.26, 2.7, and 2.47 dropped approximately 250-fold compared to the exponential phase. Expression level in pSEVA221-*DSD*-1.77 was continuously low during the cultivation, approximately 10^8^-fold lower than in pCDFDuet-*DSD*-T7, and 1000-fold lower than the “promoterless” plasmid (pSEVA221-*DSD-*No_promoter).

Note that *E. coli* ATCC 31182 lacks the T7-RNAP and transcription by the T7-promoter is not expected. However, PCA production and *DSD* expression were detected in this strain when transformed with pCDFDuet-*DSD*-T7 (Figs. 4 and 6). *E. coli* ATCC 31882 was chosen in previous studies since it has been engineered to overproduce phenylalanine and gave twofold higher PCA yield from glucose compared to BL21(DE3), a common expression host that has the T7-RNAP (Örn et al. 2021). However, Fig. 3 and Table 2 show that *DSD* is expressed even in the absence of T7-promoter, albeit at a lower level of expression—240-fold lower at 18 h compared to that with the T7-promoter (Fig. 6). On the other hand, removal of the promoter upstream of *DSD* in the SEVA221 plasmid resulted in a steeper drop, equivalent to approximately 47,000-fold lower in the mid-exponential phase (18 h).

The average of all the measured *C*_q_ values can be found in Table S2, and all the calculated normalized *C*_q_ values (ΔC_q_), relative *C*_q_ between the T7 and synthetic promoters (ΔΔC_q_), and the fold change in expression (2^−ΔΔCq^) are listed in Table S3.

SDS-PAGE of soluble and insoluble protein fractions of ATCC 31882 pCDFDuet-*DSD*-T7 and pSEVA221-*DSD*-2.7, 1.26, or 2.47 cultivated in shake flasks did not show any visible band of DSD protein at 24 h or 48 h. Similar results were observed with the specific production of PCA where the production rates are higher in strains with the synthetic promoters compared to the T7 promoter (Fig. S1, Table S4).

## Discussion

Three novel constitutive promoters were generated using a degenerate synthetic promoter approach giving enhanced transcription of the *DSD* gene for PCA production in *E. coli*. The highest increase in the PCA yield from glucose of up to 21% was observed with the synthetic promoter 1.26, compared to the previously used strain ATCC 31882 pCDFDuet-*DSD*-T7 using the T7-promoter (Örn et al. 2021). Intriguingly, the PCA production pattern by the strains using the three synthetic promoters was similar despite a slight variation in gene expression levels. This could in part be explained by the level of expressed activity of DSD, i.e., above a certain expression level, differences in enzyme concentration would have little effect on the rate and amount of PCA produced. A similar effect may be perceived in case the concentration of 3-dehydroshikimic acid (the substrate for DSD) formed in the cells is the rate limiting factor, in which case the reaction would not proceed irrespective of the level of enzyme activity. The acetate formation, which we have previously noted to be induced during the production of PCA or even when PCA was added externally (Örn et al. 2021), was two times higher in *E. coli* ATCC 31882-pSEVA221-*DSD*-1.26, 2.7, and 2.47 (harboring constitutive promoters) compared to *E. coli* ATCC 31882 and ATCC 31882-pCDFDuet-*DSD*-T7 (Table 2). This may imply a higher level of stress, which eventually leads to activation of the pathway for anaerobic metabolism to acetate in the strains with synthetic promoters. On the other hand, a significant amount of glucose is converted into CO_2_ in the ATCC 31882 pCDFDuet-*DSD*-T7 cells.

As noted in the “Results” section, there were promoters with promising high PCA levels but that failed to show consistent results. These variations could have different reasons. Variation in the cultivation condition between replicates, e.g., pSEVA221-DSD-1.26 in deep well plates, produced 0.3 g/L PCA, in shake flasks 1 g/L, and in bioreactor 3.5 g/L using the same medium. Therefore, it is possible that the promoters which produced PCA in all replicates are more stable expressed than the others. Another explanation could be that PCA formed is below the detection limit which is around 0.005 g/L (the lowest standard used contained 0.02 g/L PCA) in the diluted sample run on the HPLC, and all samples were diluted 10 times. As an example of this, promoter 1.42 which in replicate 1 produced 0.7 g/L PCA also had an CDW of 3.8 g/L; in the other replicates the CDW were 0.05 g/L and 0.7 g/L and no PCA was detected. Therefore, as the CDW is lower in the other replicates the expected PCA concentration would also be lower and possibly below the detection limit. The same pattern was also seen for 1.52 but not for 1.35 which had a high CDW in all replicates 3.1, 3.4, and 1 g/L. As we did not have enough evidence to determine a definitive cause for the differences, we chose to continue with those that had constant PCA production.

The RT-qPCR results suggest that the synthetic constitutive promoters generated in this work, despite having variable sequences, are regulated in the same manner. The drop in expression observed in our experiments might not be an expected behavior for a constitutive promoter, especially those recognized by σ^70^, whose expression is expected to be constant and independently regulated by transcription factors (Brewster et al. 2012; Engstrom and Pfleger 2017; Shimada et al. 2014; Westmann et al. 2018). However, suggesting that constitutive promoters are not regulated at all would be misleading and growth-related expression has been observed in σ^70^ recognized promoters (Ishihama 2000, 2009; Shimada et al. 2014), and most likely occurs with the described synthetic promoters.

The transcription of *DSD* under the synthetic promoters decreases after the PCA concentration reaches its upper limit. The PCA concentration or the resulting high levels of acetate could pose a burden for the cells and force the constitutive promoters to downregulate the expression of the *DSD* gene in the new strains. In general, gene expression is controlled in many ways, besides promoter strength (Alper et al. 2005). *Cis*- and *trans*-acting factors also affect the transcription, the former regulating the transcription of nearby genes or operons, while the *trans*-acting factors can affect distant genes by binding to their target sequences (Wittkopp et al. 2004). If PCA affects those factors or if the regulation occurs through other means is unknown but possible. Other aspects which affect global transcription regulation patterns in *E. coli*, which could also affect the expression of *DSD,* are the nutrient availability (Harman 2001; Oh et al. 2002; Ramseier 1996), oxidative stress (Zheng et al. 2001), level of acidity (Masuda and Church 2003), and growth stage (Ishihama 2000; 2009; Shimada et al. 2014).

The observed PCA production using *E. coli* ATCC 31182 (which lacks the T7-RNAP) transformed with the plasmid pCDFDuet-*DSD*-T7 containing a T7-promoter was surprising. One may speculate that a native RNAP recognizes the T7-promoter or another promoter sequence on the plasmid that is not terminated correctly and continues to transcribe the plasmid. To elucidate the functionality of the transcription regulation elements of the pCDFDuet plasmid in strains that possess or lack the T7-RNAP, plasmids with altered regulatory elements were constructed. In all cases with the pCDFDuet vector, the removal of 5′UTR or the addition of terminators in the presence of IPTG showed reduction in the PCA production levels. Likewise, with pSEVA221 vector, the removal of the degenerate promoter or the addition of the potential promoter detected by iPromoter-2L revealed low levels of PCA.

Altogether, the results indicate background PCA levels (approx. 0.04 g/L) in the absence of a transcriptional regulatory element. The presence of another detected promoter sequence (pSEVA221-*DSD*-Duet_promoter) did not lead to any increase in PCA production. Furthermore, we found that PCA production occurs in the pCDFDuet plasmids with and without IPTG in *E. coli* ATCC 31882, BL21(DE3) and BW25113, at different concentrations. This data suggests that there is most likely another region that initiates transcription of *DSD* in both the pSEVA221 and pCDFDuet plasmid, which could not be revealed in this study*.* The basal production could however be reduced in the pCDFDuet plasmid by addition of a terminator upstream of the *DSD* gene when not adding IPTG.

The only condition at which the activity of lacO in the expression cassette was confirmed was when PCA was not produced in the non-induced *E. coli* ATCC 31882 carrying pCDFDuet-*DSD*-5′_terminator. We could also see that the T7-promoter and 5′UTR of pCDFDuet, on their own, were able to produce PCA in strains lacking the T7-RNAP. Furthermore, strains with the complete T7 promoters produced some PCA even when IPTG was not added. Altogether, this tells us that the T7 system is not tightly regulated and lacks specificity (“*leaky T7*”). Even though its use is widespread, its basic and detailed mechanism of action is unknown and requires further studies to understand it fully. However, it seems unlikely that the expression of *DSD* in the pCDFDuet plasmid is an isolated ORF as the introduction of the suspected promoter sequence to the pSEVA221 plasmid did not increase the PCA levels beyond the production of 0.04 g/L like pSEVA221-*DSD*-No_promoter. Whether the T7-promoter should be considered only as a portion of the UTR of a larger mRNA could not be determined in this study. However, all other genes on the pCDFDuet plasmid are expressed in the antisense direction compared to *DSD* which means if another categorized promoter is used (i.e., *lacI* or *SmR*), it needs to be bidirectional, a feature only recently described in *E. coli* (Warman et al. 2021).

To conclude, this study shows that the use of synthetic constitutive promoters can overcome the drawbacks of the T7-expression system, such as the decrease in the overexpression levels over a long time due to chromosomal mutation (Vethanayagam and Flower 2005), and the need for the costly inducer IPTG. Also, there are examples where the protein overexpression inhibits the production of a compound of interest, as shown for lycopene and (R, R)-2,3-butanediol overproduction (Ji et al. 2015; Rodríguez-Villalón et al. 2008; Yoon et al. 2006; Zhou et al. 2011) (Fig. 3). While native and synthetic constitutive promoters can be used as an alternative to commercial inducible promoters, selecting the correct promoter is important, and it is not possible to know in advance how the expression of a novel promoter will be regulated. Discoveries of bidirectional promoters in bacteria and eukaryotes (Preker et al. 2008; Warman et al. 2021) and transcription of antisense DNA in eukaryotes (He et al. 2008; Neil et al. 2009) challenge long considered views (Browning and Busby 2016). Even a well-characterized expression system like the pCDFDuet is not fully understood.

Our results show that despite having different transcription levels, the three synthetic promoters did not significantly affect the growth or production profiles of PCA; in fact, the PCA yield with one of the promoters was significantly increased compared to the induced pCDFDuet plasmid. Furthermore, the novel synthetic promoters yielded a high level of expression even when a low copy plasmid (i.e., pSEVA221) was used (copy number of 1–2) (Martínez-García et al. 2015) to mimic the condition in which the *DSD* gene would be integrated into the chromosome to create cells with a stable genetic modification. The full scope of the plasmid library, which reaches over 10^12^ unique combinations, was not examined in this study. The use of high-throughput automation should increase the probability of finding other promising promoter sequences with higher efficiency than those found in the present study, where only 180 colonies were analyzed. Nevertheless, three hits were identified with higher PCA yield than that produced by T7-promoter controlled system.

## Supplementary Information

Below is the link to the electronic supplementary material.

Supplementary file1 The supplementary material includes the list of primers used in the study, PCA production in E. coli BL21(DE3) and BW25113, and additional data on the expression studies. (PDF 459 KB)

## Data Availability

All data obtained in the study is included in the paper and its online Supporting Information. The datasets used and/or analyzed during the current study are available from the corresponding author on request.
