# Supplementary material for: Enhancing metabolic efficiency via novel constitutive promoters to produce protocatechuic acid in Escherichia coli
Source: Appl Microbiol Biotechnol. 2024 Aug 17;108:442. doi: 10.1007/s00253-024-13256-6 (PMC11330383; doi:10.1007/s00253-024-13256-6)
Supplement: Supplementary file 1 [file 253_2024_13256_MOESM1_ESM.pdf]

Applied Microbiology and Biotechnology

Enhancing Metabolic Efficiency via Novel Constitutive Promoters to Produce Protocatechuic Acid in *Escherichia coli*

Oliver Englund Örn, Arne Hagman, Mohamed Ismail, Nélida Leiva Eriksson*, Rajni Hatti-Kaul*

Division of Biotechnology, Department of Chemistry, Centre for Chemistry and Chemical Engineering, Lund University, Lund, Sweden

***Corresponding authors**

E-mail: nelida.leiva_eriksson@biotek.lu.se (NLE); Tel.: +46 46 2228193; ORCID: 0000-0001-7712-4683.

E-mail: Rajni.Hatti-Kaul@biotek.lu.se (RHK); Tel.: +46 46 2224840; ORCID: 0000-0001-5229-5814

Table S1. List of all primers used in this work and the probable Duet promoter sequence.

Amplicon	Forward primer	Reverse primer	Amplicon length	Reference
**Degenerate promoter**	AGGCCGCCTAGGCCG	CAAGGACACGGTAGCGATCGAACG	191 bp	This work
***DSD* gene**	ATGCAGCGTTCGATCGC	CTACAGCACCGGCTTGC	1908 bp	This work
***B0015* Terminator**	AGCGGGGCGGCGCGCAAGCCGGTGCTGTAGACACCTGCAATGCATGAGCTCGCATGCCCA	ACACAAATTTAAATCGTAATTATTGGGGACCCCCAAGCTTGCCGGCTAGTAACATCTCAC	246 bp	This work
**Sequencing primer**	CTCCTGCATCAGGTCGAACTT			This work
***DSD* cDNA**	CTTATGACGGCGTGGAGTTC	AGGAATACGGTTCGGCGTTC	174 bp	This work
***cysG* cDNA**	TTGTCGGCGGTGGTGATGTC	ATGCGGTGAACTGTGGAATAAACG	105 bp	Zhuo et al. 2011
***hcaT* cDNA**	GCTGCTCGGCTTTCTCATCC	CCAACCACGCTGACCAACC	86 bp	Zhuo et al. 2011
***idnT* cDNA**	CTGTTTAGCGAAGAGGAGATGC	ACAAACGGCGGCGATAGC	90 bp	Zhuo et al. 2011
Predicted Duet promoter (iPromoter-2L)	GACGCTCTCCCTTATGCGACTCCTGCATTAGGAAATGGAATTGTGAGCGGATAACAATTCCCCTGTAGAAATAATTTTGTTTAACTTTAATAAGGAGATATACC			This work

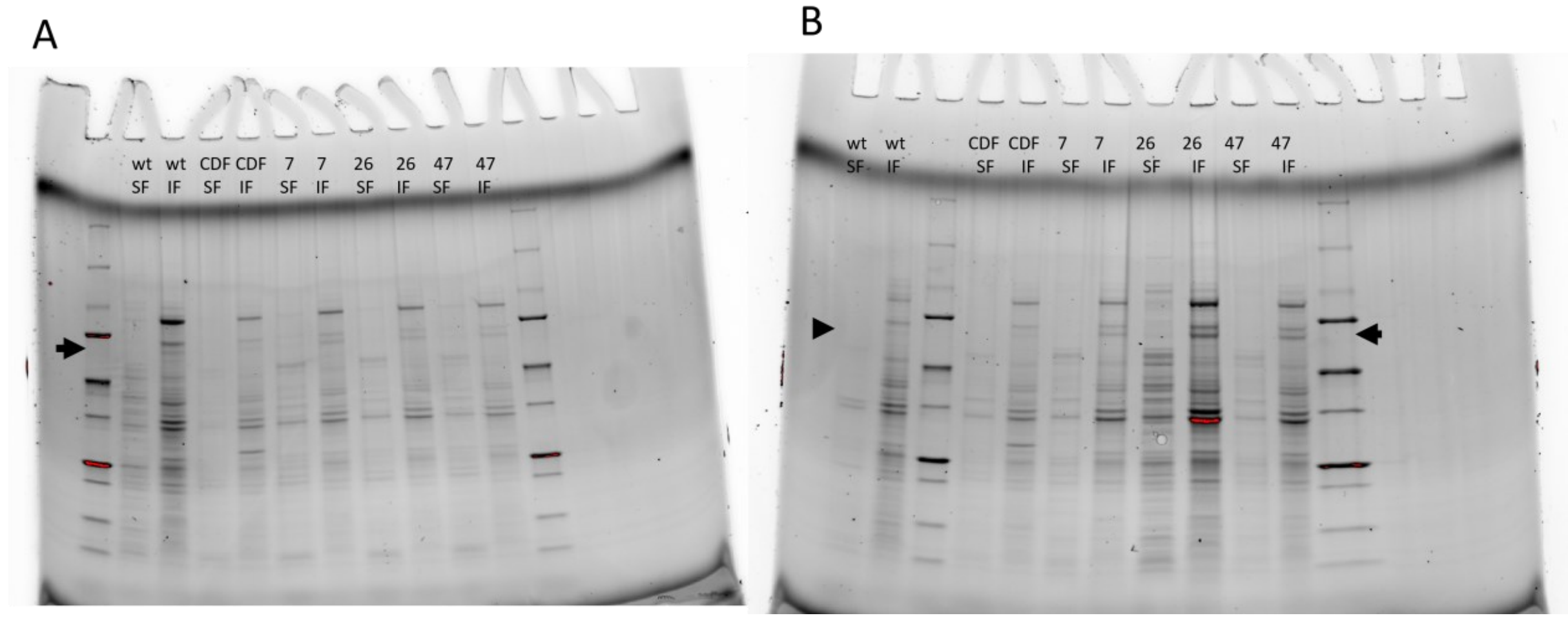

Figure S1. SDS-PAGE of soluble (SF) and insoluble (IF) protein fractions of cells at (a) 24 h and (b) 48 h from a shake flask cultivation of *E. coli* ATCC 31882 without DSD gene (wt), with inducible DSD expression (CDF), and constitutive DSD expression (7, 26 and 47). DSD has a molecular mass of 70 kDa but it cannot be detected in the gel. Precision Plus Protein Unstained Protein Standards (Biorad) were used to determine molecular wight of protein bands and the black arrows indicate the expected position of DSD at 70 kDa. The samples and gels were prepared together and run in parallel. The pictures were taken separately with a GelDoc Go Imaging System (Biorad) and visualised in the Image Lab Touch Software (Biorad).

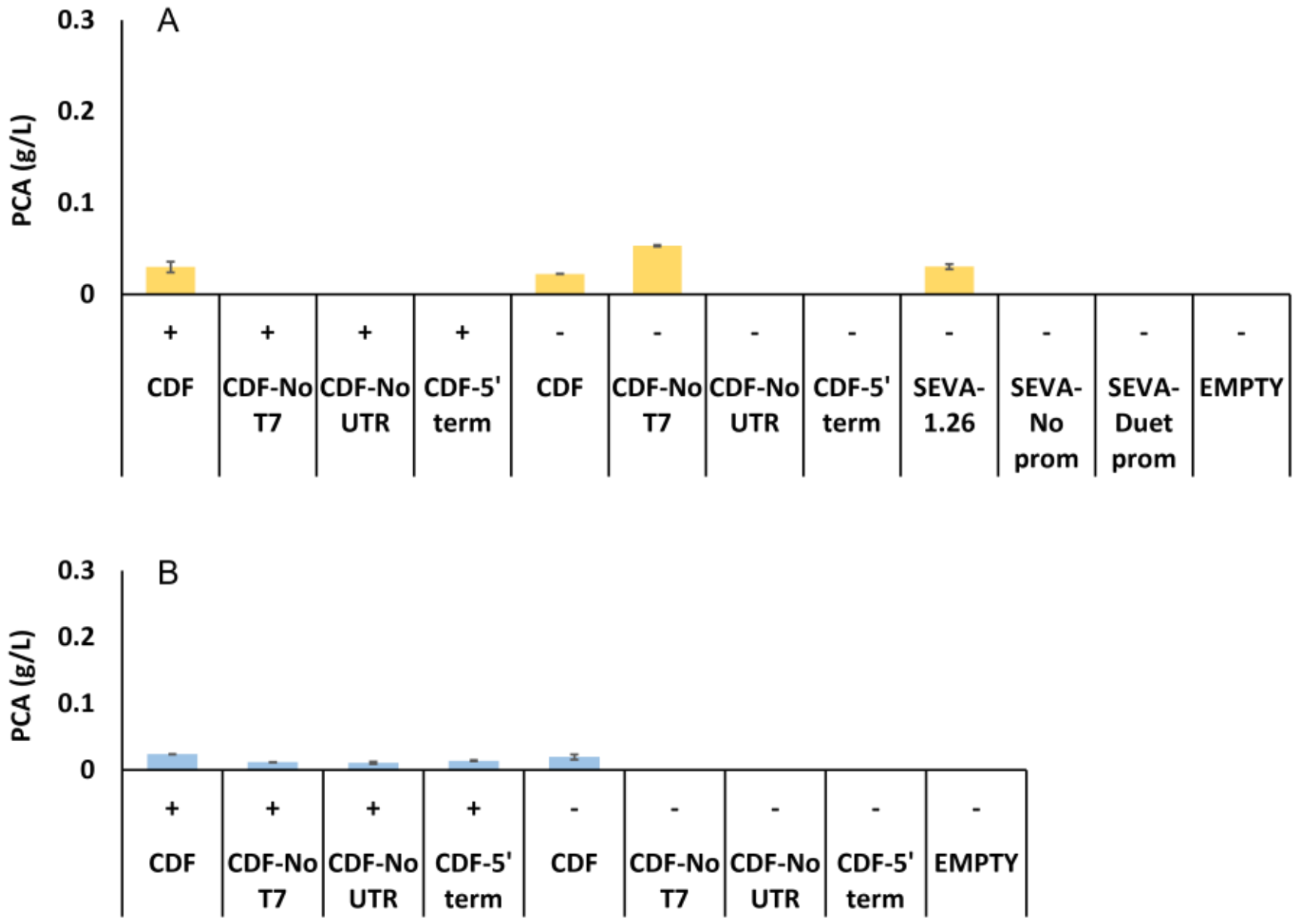

Figure S2. PCA titres in the 48 h culture in A) *E. coli* BW25113 and B) *E. coli* BL21(DE3). The cells were transformed with the plasmids, pCDFDuet-DSD (CDF); pCDFDuet-DSD-No_T7 (CDF-No T7); pCDFDuet-DSD-No_UTR (CDF-No_UTR); pCDFDuet-DSD-5'_terminator (CDF-5'_term); pSEVA221-DSD-1.26 (SEVA-1.26); pSEVA221-DSD-No_promoter (SEVA-No prom); and pSEVA221-DSD-Duet_promoter (SEVA-Duet prom), respectively. The cells were grown in 1 mL modified M9 medium in a 96-deepwell plate in the presence (+) or absence (–) of 1 mM IPTG in the cultivation medium. All strains were cultivated in triplicates and error bars represent the standard deviation.

Table S2. C_q values at 18h, 24h and 48h of *DSD*, *cycG*, *hcaT* and *idnT* genes in the *E. coli* strain ATCC 31882 transformed with pCDFDuet-DSD, pSEVA221-DSD 2.7, pSEVA221-DSD 1.26, SEVA221-DSD 2.47, pSEVA221-DSD-1.77, ATCC 31882- pCDFDuet-DSD-No_T7 and ATCC 31882-pSEVA221-DSD-No_promoter.

		**ATCC (C_q)**	**pCDF Duet-*DSD* (C_q)**	**pSEVA 221-*DSD*-2.7 (C_q)**	**pSEVA 221-DSD-1.26 (C_q)**	**pSEVA 221-DSD-2.47 (C_q)**	**pSEVA 221-DSD-1.77 (Cq)**	**pCDF Duet-DSD-No_T7 (Cq)**	**pSEVA 221-DSD-No_prom (C_q)**
**18 h**	DSD		21.07±0.36	19.19±0.37	19.82±0.26	21.04±1.14	31.66±2.32	14.53±2.70	23.50±0.37
	CycG	29.03±1.86	29.84±0.76	29.29±0.42	28.32±0.14	28.85±0.62	21.05±0.43	22.47±0.87	21.24±0.85
	hcaT	26.87±0.94	29.33±0.34	29.60±0.32	29.78±0.80	30.83±1.36	25.15±0.32	23.96±1.08	23.83±0.31
	idnT	28.88±1.02	30.66±0.33	29.26±0.39	29.85±0.49	30.34±1.86	22.73±0.47	24.53±1.17	23.29±0.37
**24 h**	DSD		16.55±0.62	15.98±0.30	15.87±0.19	16.20±1.27	34.21±3.32	17.65±0.73	20.47±0.59
	CycG	29.03±1.86	29.84±0.76	29.29±0.42	28.32±0.14	28.85±0.62	23.11±0.60	21.67±0.46	22.72±2.74
	hcaT	23.57±0.54	25.99±0.59	24.04±0.58	24.67±0.26	25.32±0.70	22.40±0.73	24.58±1.00	23.90±0.39
	idnT	26.50±0.24	27.35±1.06	24.86±0.33	25.26±0.80	26.26±0.55	24.74±0.67	24.87±0.45	24.47±0.66
**48 h**	DSD		20.57±0.49	27.15±0.38	26.77±0.51	26.21±0.82	34.68±1.00	21.47±0.46	26.25±0.18
	CycG	29.03±1.86	29.84±0.76	29.29±0.42	28.32±0.14	28.85±0.62	22.49±0.27	26.29±0.40	23.16±0.49
	hcaT	29.63±0.65	32.64±0.77	32.13±1.08	30.52±1.19	30.11±0.47	25.17±0.16	30.62±0.90	27.15±0.22
	idnT	29.56±0.90	33.07±1.41	32.15±0.45	31.87±0.80	30.07±0.58	26.25±0.30	31.43±1.37	27.78±0.29

Table S3. Relative expression-change of DSD gene by the three synthetic promoters compared to the expression in pCDFDuet-DSD by the T7-promoter in *E. coli* ATCC 31882.

		**pCDF Duet-DSD**	**pSEVA 221-DSD 2.7**	**pSEVA 221-DSD 1.26**	**pSEVA 221-DSD 2.47**	**pSEVA 221-DSD 1.77**	**pCDF duet-DSD no-T7 promoter**	**pSEVA 221-DSD no-promoter**
**18h**	Normalized Cq (ΔCq)	-7.99± 0.41	-8.85± 0.31	-8.53± 0.32	-8.32± 0.31	14.17± 2.28	-1.87± 2.43	7.12± 0.48
	Relative Cq vs CDF (ΔΔCq)		-0.86± 0.24	-0.54± 0.34	-0.33± 0.30	25.56± 2.45	9.41± 2.49	18.34± 0.50
	Fold change in expression ($2^{-\Delta\Delta Cq}$)		1.83± 0.30	1.50± 0.36	1.28± 0.26	5.28E-08± $4.88\cdot10^{-8}$	4.16E-03± $4.00\cdot10^{-3}$	3.18E-06± $9.26\cdot10^{-7}$
**24h**	Normalized Cq (ΔCq)	-9.60± 0.44	-8.04± 0.33	-8.53± 0.41	-8.88± 0.94	8.79± 1.74	-9.52± 3.05	0.55± 0.27
	Relative Cq vs CDF (ΔΔCq)		1.56± 0.49	1.07± 0.56	0.73± 1.06	22.75± 3.10	4.88± 0.58	11.36± 0.88
	Fold change in expression ($2^{-\Delta\Delta Cq}$)		0.36± 0.12	0.51± 0.18	0.76± 0.45	$5.03\cdot10^{-7}$± $4.95\cdot10^{-7}$	0.0370± 0.016	$4.50\cdot10^{-4}$± $2.42\cdot10^{-4}$
**48h**	Normalized Cq (ΔCq)	-11.28± 0.45	-4.04± 0.51	-3.47± 1.07	-3.47± 0.42	10.00± 0.93	-7.98± 0.84	0.27± 0.23
	Relative Cq vs CDF (ΔΔCq)		7.24± 0.65	7.81± 1.13	7.81± 0.69	21.11± 0.93	3.30± 0.96	11.55± 0.61
	Fold change in expression ($2^{-\Delta\Delta Cq}$)		$7.24\cdot10^{-3}$± $2.87\cdot10^{-3}$	$5.83\cdot10^{-3}$± $3.82\cdot10^{-3}$	$5.00\cdot10^{-3}$± $2.47\cdot10^{-3}$	$5.15\cdot10^{-7}$± $2.19\cdot10^{-7}$	0.13± 0.077	$3.63\cdot10^{-4}$± $1.52\cdot10^{-4}$

Table S4. Specific production of PCA. Production rates are higher in strains with the synthetic promoters compared to the T7 promoter.

**Strain**	**Max PCA production rate (1/h) (12-18h)**
ATCC 31882+pCDFDuet-DSD-T7	0.3022
ATCC 31882 pSEVA-DSD-2.7	0.8845
ATCC 31882 pSEVA-DSD-1.26	0.7257
ATCC 31882 pSEVA-DSD-2.47	0.6207
BL21(DE3)+pCDFDuet-DSD-T7	0.7009

Reference

Zhou K, Zhou L, Lim QE, Zou R, Stephanopoulos G, Too H.-P. (2011) Novel reference genes for quantifying transcriptional responses of *Escherichia coli* to protein overexpression by quantitative PCR. BMC Mol. Biol. 12**,** 1-9. doi: 10.1186/1471-2199-12-18.
